# Efficient reverse water gas shift reaction at low temperatures over an iron supported catalyst under an electric field

**DOI:** 10.1038/s41598-024-61017-2

**Published:** 2024-05-03

**Authors:** Masaki Yamaoka, Keidai Tomozawa, Koki Sumiyoshi, Tadaharu Ueda, Shuhei Ogo

**Affiliations:** 1 Department of Marine Resources Science, Faculty of Agriculture and Marine Science, Kochi University, Nankoku, Kochi 783-8502 Japan; 2 Marine Core Research Institute, Kochi University, Nankoku, Kochi 783-8502 Japan; 3 MEDi Center, Kochi University, Kochi, 780-0842 Japan

**Keywords:** Heterogeneous catalysis, Green chemistry

## Abstract

The development of high-performance Fe-based catalysts is attractive because Fe is a cost-effective and earth-abundant element. Application of an external electric field and an appropriate catalytic support to an Fe-based catalyst enabled the reverse water–gas shift reaction to proceed with high activity, selectivity, and durability even at the low temperature of 423 K. The Fe-supported catalyst showed superior CO selectivity (≈ 100%) compared to the Co- or Ni-supported catalyst. The apparent activation energy (5.9 kJ mol^−1^) over the Fe/Ce_0.4_Al_0.1_Zr_0.5_O_2_ catalyst under an electric field was much lower than that without an electric field (61.4 kJ mol^−1^).

## Introduction

Extensive attention has been given to solving the important issue that anthropogenic emissions of greenhouse gases such as CO_2_ could accelerate global warming. Recently, a variety of CO_2_ capture and utilization (CCU) technologies have been developed because they are regarded as effective ways to reduce artificial CO_2_ emissions^1–3^. Catalytic conversion of CO_2_ with green H_2_ produced by electrolysis of water using renewable energies is a promising candidate for CCU technology^4^. Many methods for converting CO_2_ into valuable chemicals, such as CH_4_, CO, and CH_3_OH, have been reported^4–12^. In particular, CO production from CO_2_ through the reverse water–gas shift (RWGS) reaction (Eq. 1) is quite important because CO can be further converted into high-value chemicals and fuels, such as hydrocarbons and various oxygenates, via Fischer–Tropsch (FT) synthesis or well-established industrial processes, respectively^4,13,14^, which is mainstream for C1 chemistry.

1 $${{\text{CO}}}_{2}+ {{\text{H}}}_{2}\leftrightarrows {\text{CO}}+{{\text{H}}}_{2}{\text{O}},\Delta {H}_{298}^{^\circ }=41.2\;{\text{kJ}}/{\text{mol}}$$

However, high-temperature heating is required to obtain high CO_2_ conversion due to the thermodynamic equilibrium constraints for the RWGS reaction, leading to many problems in terms of energy and catalyst durability. Therefore, CO_2_ conversion at low temperatures requires unconventional catalytic reaction techniques. In particular, the application of an external direct current electric field to metal-supported metal oxide semiconductor catalysts has enabled several catalytic reactions^15,16^ that include CO_2_ activation, such as the RWGS reaction^17,18^, CO_2_ methanation^5^ and dry reforming of methane^19,20^, to proceed even below 473 K. Both surface hydrogen migration and redox reactions that use lattice oxygen vacancies of metal oxide supports promoted by an electric field have resulted in the RWGS reaction proceeding smoothly and selectively with redox reactions even at low temperatures over Ru-supported catalysts^18^.

Highly active and selective catalysts for the RWGS reaction have been extensively developed^21^ based on various precious metal-supported materials (e.g., Pt/La-ZrO_2_^17^, Rh/Fe-CeO_2_^22^, Ru/ZrTiO_4_^18^, PtMn/SiO_2_^23^) and base metal materials (e.g., Fe-based^24^, Ni-based^25^, Cu-based catalysts^26^). Considering the amount and price of metal resources, Fe-based catalysts should be preferred for practical use. However, preparing stable high-performance Fe-based catalysts with high Fe dispersion is difficult because the supported Fe particles easily agglomerate during catalyst preparation, prereduction and/or catalytic reactions. On the other hand, the anchoring effect of Al doped in an oxide support effectively suppresses agglomeration of the supported Fe particles^27^. Such highly dispersed Fe catalysts could exhibit high activity and stability in low-temperature RWGS reactions under an electric field. In this study, high-performance Fe-supported catalysts were developed by controlling the interaction between support oxides and supported Fe for use in the RWGS reaction under an electric field.

## Results and discussion

**Figure 1:**
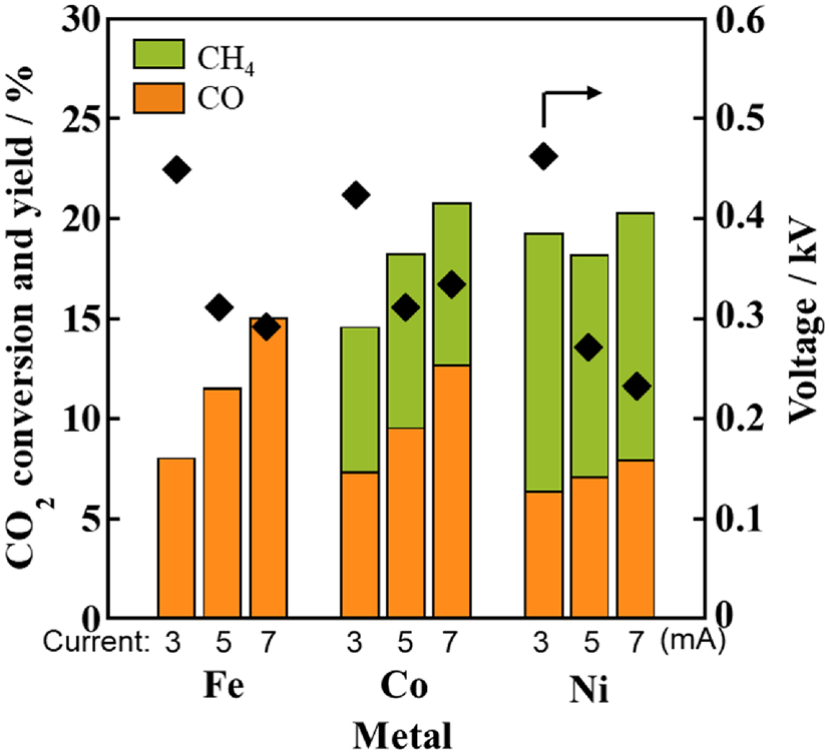
CO_2_ conversion and product yields in the RWGS reaction over various 10 wt% metals supported on Ce_0.4_Al_0.1_Zr_0.5_O_2_ at 423 K under an electric field. Furnace temperature: 423 K; catalyst weight: 100 mg; input current (mA): 3.0, 5.0, and 7.0; gas composition (%): CO_2_:H_2_:Ar = 25:25:50; total gas flow rate: 100 mL min^−1^.

Catalytic activity tests for the RWGS reaction under an electric field were conducted with 10 wt% Fe, Co, or Ni supported on Ce_0.4_Al_0.1_Zr_0.5_O_2_ (CAZO) to elucidate the effects of the supported metal on the catalytic activity and selectivity (Fig. 1). All of the tested M/CAZO catalysts showed catalytic activity even at the low temperature of 423 K. In particular, the Fe-supported catalyst exhibited higher CO selectivity (ca. 100%), although its catalytic activity was slightly lower, while the Co- and Ni-supported catalysts exhibited high CH_4_ selectivity. The CO_2_ conversion over the Fe- and Co-supported catalysts increased with increasing applied current, while that over the Ni-supported catalyst was independent of the applied current. Clearly, the supported metal affected the catalytic activity and selectivity, and the Fe-supported catalyst was suitable for obtaining CO from the RWGS reaction with high selectivity.

**Figure 2:**
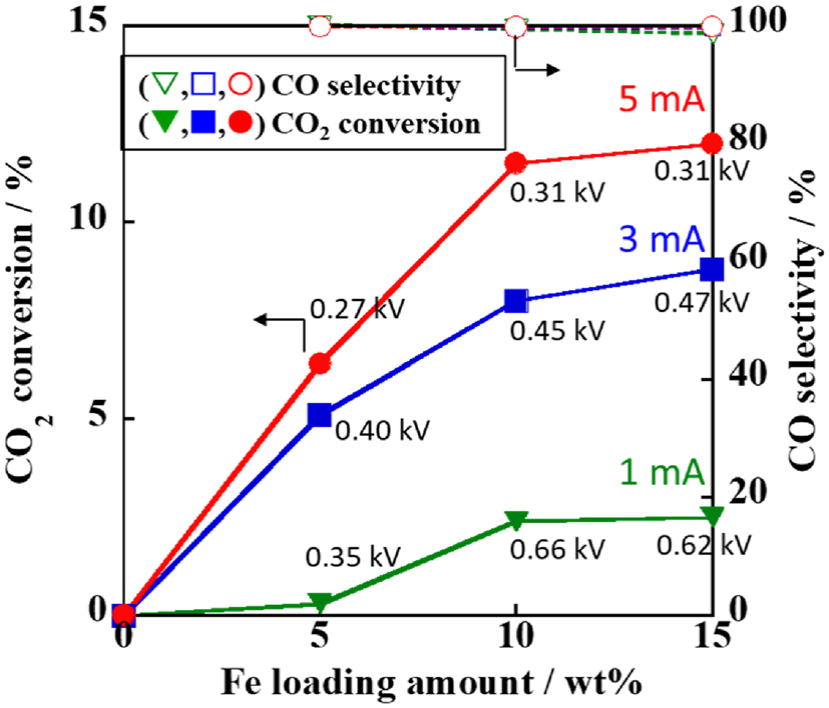
Effect of the Fe loading amount on the CO_2_ conversion and CO selectivity in the RWGS reaction over the Fe/CAZO catalysts under an electric field at various input currents. Furnace temperature: 423 K; catalyst weight: 100 mg; input current (mA): (green filled triangles) 1.0, (blue filled squares) 3.0, and (red filled circles) 5.0; gas composition (%): CO_2_:H_2_:Ar = 25:25:50; total gas flow rate: 100 mL min^−1^.

The catalytic activity was investigated by using the Fe-supported CAZO catalysts with various Fe loading amounts to clarify the effect of the Fe loading amount on the RWGS activity under an electric field at various input currents (Fig. 2). The CAZO support without Fe loading showed no catalytic activity. The CO_2_ conversion increased with increasing Fe loading up to 10 wt% and then became constant. Although the number of active sites was sufficiently large at 10 wt% Fe, the activity increased proportional to the input current. The catalytic activity depends on both the Fe loading amount and the input current. Moreover, these Fe-supported catalysts under applied 1–5 mA currents exhibited nearly 100% CO selectivity (Fig. 2).

**Figure 3:**
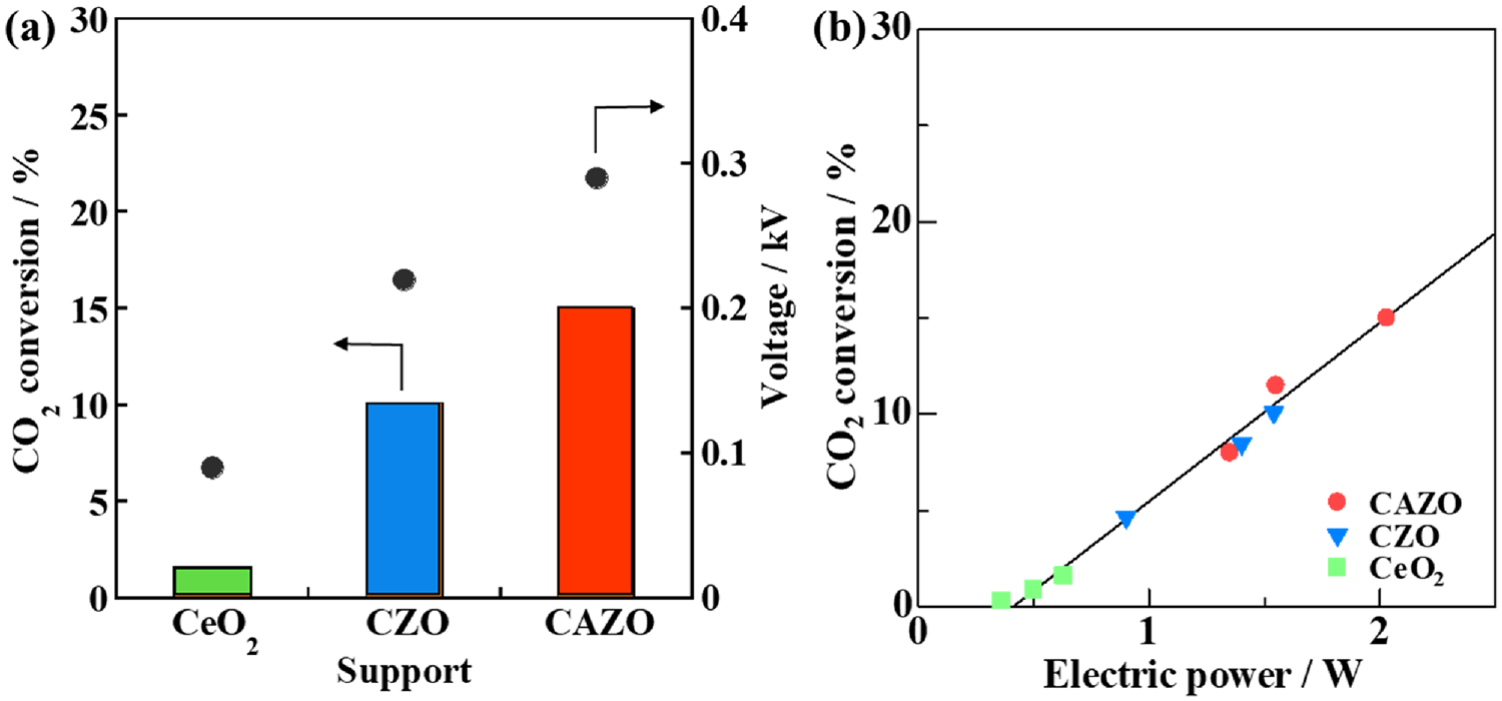
(**a**) Effects of the support on the CO_2_ conversion and response voltage and (**b**) effect of the input electric power on the CO_2_ conversion for the RWGS reaction over 10 wt% Fe-supported catalysts at 423 K under an electric field. Furnace temperature: 423 K; catalyst weight: 100 mg; input current (mA): 3.0–7.0; gas composition (%): CO_2_:H_2_:Ar = 25:25:50; total gas flow rate: 100 mL min^−1^.

The catalytic activity was also investigated by using Fe-supported catalysts with different supports, such as CeO_2_, Ce_0.5_Zr_0.5_O_2_ (CZO) or CAZO, to clarify the effect of the support on the catalytic RWGS activity under an electric field. All three Fe-supported catalysts under an applied 3–7 mA current exhibited nearly 100% CO selectivity (Fig. 3), and the highest CO_2_ conversion was observed for the Fe/CAZO catalyst. The CO_2_ conversion was correlated with the response voltage because of the galvanostatic control. The CO_2_ conversion increased with the applied electric power (Fig. 3b), independent of the catalytic support, implying that the promoting effects of the electric field is almost the same for the three Fe-supported catalysts.

By applying Scherrer’s equation, the crystallite sizes of the supported Fe were calculated to be d = 27.5, 36.7 and 27.5 for Fe/CeO_2_, Fe/CZO, and Fe/CAZO, respectively, based on the X-ray diffraction (XRD) patterns of the three Fe-supported catalysts (Fig. S1). The catalytic activity was independent of the crystallite size, although the crystallite size of Fe/CZO was slightly larger than that of the other catalysts. The support affected the electronic and/or ionic conductivity rather than the particle size of the supported Fe, indicating that Fe/CAZO, for which a higher voltage (power) could be applied, exhibited higher catalytic activity.

**Figure 4:**
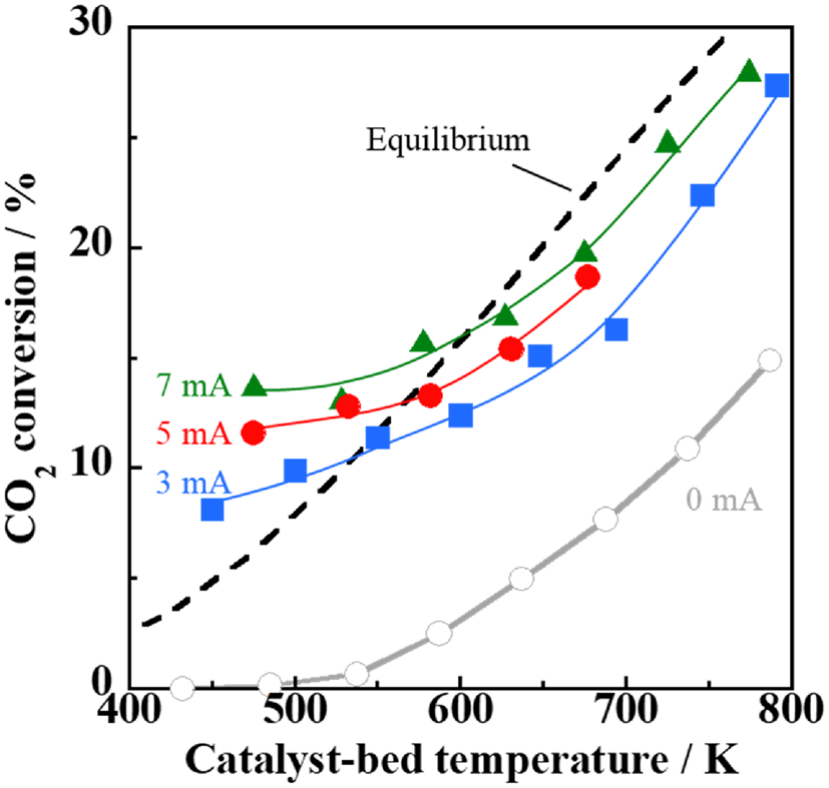
Temperature dependence of the CO_2_ conversion in the RWGS reaction over the Fe/CAZO catalyst under an electric field with various applied currents. Furnace temperature: 423–773 K; catalyst weight: 100 mg; input current (mA): (grey unfilled circles) 0, (blue filled squares) 3, (red filled circles) 5, (green filled triangles) and 7; gas composition (%): CO_2_:H_2_:Ar = 25:25:50; total gas flow rate: 100 mL min^−1^.

The catalytic activity for the RWGS reaction was investigated at the designated catalyst bed temperature under different powers of the electric field generated by various applied currents (Fig. 4). The black line indicates the equilibrium conversion of the RWGS reaction at CO_2_:H_2_ = 1:1. Under an electric field, the CO_2_ conversion exceeded the equilibrium conversion in the low-temperature region below 550 K, while no catalytic activity was observed in the conventional RWGS reaction without an electric field (0 mA). The catalytic activity of the Fe/CAZO catalysts under an electric field below 550 K was comparable to that of the Ru-supported catalysts^18^. When a higher current was applied, more CO_2_ was converted at the same catalyst bed temperature. The actual catalyst bed temperature, which was measured using a thermocouple, increased by 30–50 K due to Joule heating. The RWGS reaction proceeded even at low catalyst bed temperatures such as 475 K (423 K as the external temperature) when an electric field (5 mA) was applied, while no RWGS reaction proceeded at the same temperature without an electric field. Therefore, in the lower temperature region at approximately 500 K, Joule heating did not affect the catalytic activity under an electric field.

**Figure 5:**
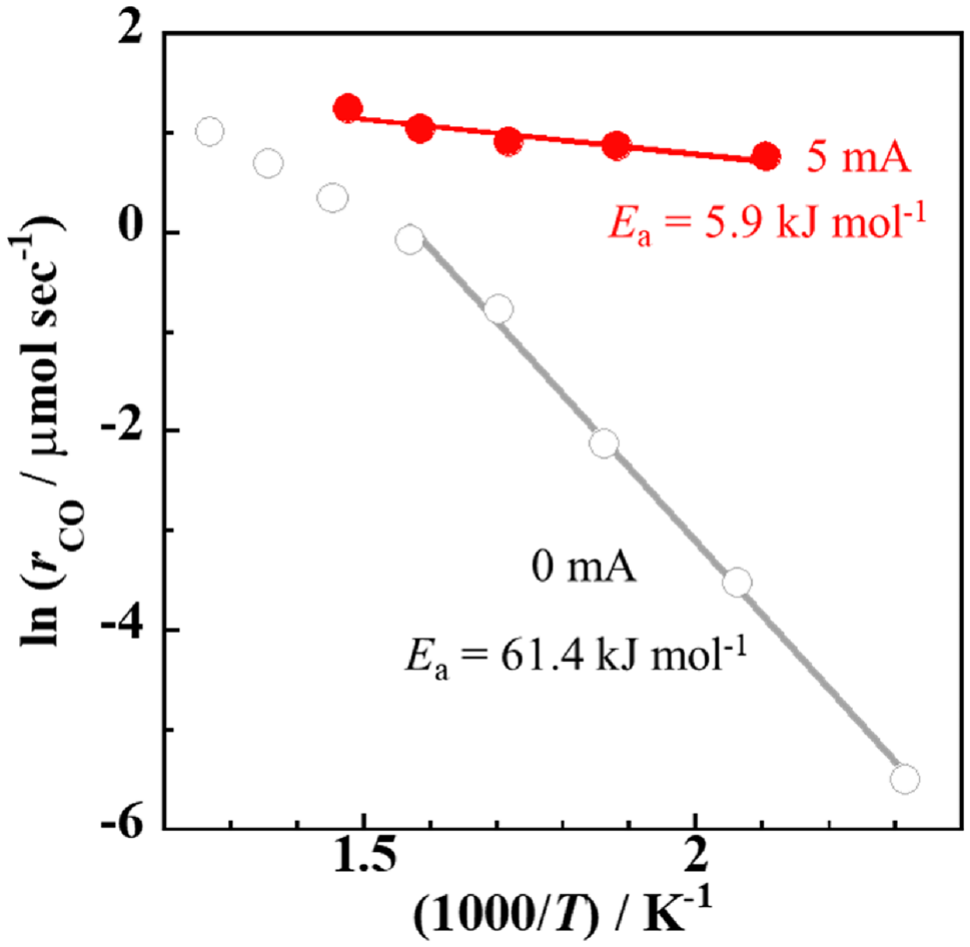
Arrhenius plots for the RWGS reaction over the Fe/CAZO catalyst with or without an electric field. Furnace temperature: 423–773 K; catalyst weight: 100 mg; input current (mA): (grey unfilled circles) 0 and (red filled circles) 5; gas composition (%): CO_2_:H_2_:Ar = 25:25:50; total gas flow rate: 100 mL min^−1^.

From the Arrhenius plots for the RWGS reaction over the Fe/CAZO catalyst (Fig. 5), the apparent activation energy was estimated to be *E*_a_ = 5.9 kJ mol^−1^ under an electric field, which was much lower than the value of *E*_a_ = 61.4 kJ mol^−1^ without an electric field. The difference in the apparent activation energy indicates that the reaction mechanism of the RWGS reaction under an electric field should be different from that of the conventional catalytic RWGS reaction without an electric field. Recently, the RWGS reaction over a Ru/ZrTiO_4_ catalyst under an electric field (*E*_a_ = 6.74 kJ mol^−1^) was reported to proceed through a redox mechanism based on in situ diffuse reflectance infrared Fourier transform spectroscopy measurements, in which the electric field promoted the formation of lattice oxygen vacancies on the surface of the metal oxide support to reduce CO_2_ to CO using the formed surface lattice oxygen vacancies even at low temperatures^18^. A periodic operation test was conducted on the Fe/CAZO catalyst under an electric field at 423 K (see the Supporting Information), and the results also supported this redox reaction mechanism. The observation that CO was produced when CO_2_ was supplied but not when H_2_ was supplied (Fig. S2) indicates that the RWGS reaction over the Fe/CAZO catalyst under an electric field could proceed through a redox mechanism similar to that for the reported Ru/ZrTiO_4_ catalyst system. Moreover, the observation that the amount of CO formed during CO_2_ supply without an electric field was significantly reduced (Fig. S3) indicates that an electric field must promote the redox reaction involving lattice oxygen vacancies.

**Figure 6:**
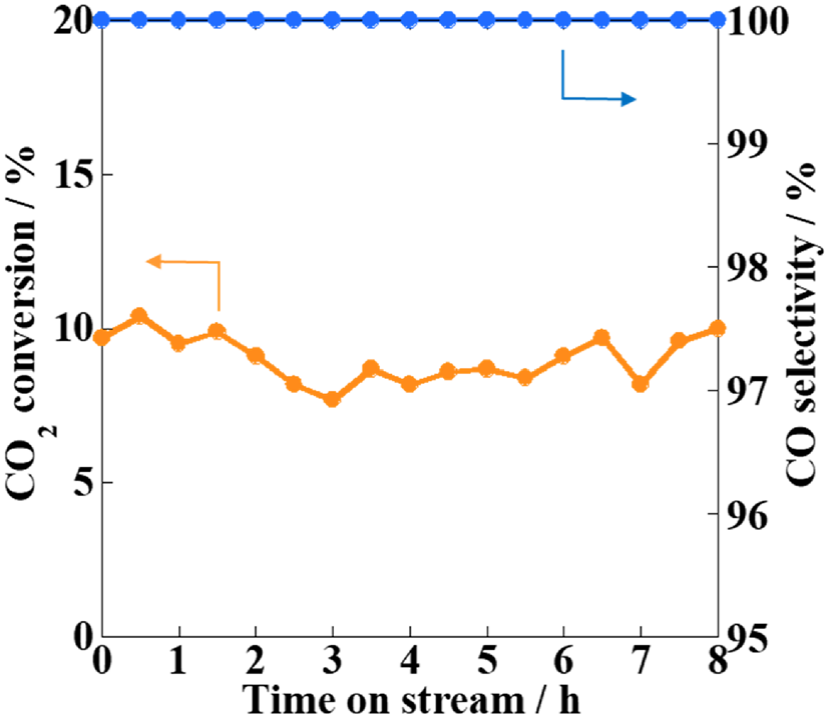
Catalytic stability during the RWGS reaction over the Fe/CAZO catalyst under an electric field. Furnace temperature: 423 K; catalyst weight: 100 mg; input current: 5.0 mA; gas composition (%): CO_2_:H_2_:Ar = 25:25:50; total gas flow rate: 100 mL min^−1^.

The CO_2_ conversion and CO selectivity variations with time on stream were investigated to evaluate the catalytic stability of the Fe/CAZO catalyst under an electric field (Fig. 6 and Fig. S4). The CO_2_ conversion remained constant at approximately 10%, despite some fluctuations, with the CO selectivity remaining constant at 100% for at least 8 h, indicating that the Fe/CAZO catalyst should be highly stable with high activity and selectivity for the RWGS reaction under an electric field.

## Conclusions

The catalytic performance of Fe-supported catalysts, i.e., Fe/CeO_2_, Fe/Ce_0.5_Zr_0.5_O_2_ (Fe/CZO) and Fe/Ce_0.4_Al_0.1_Zr_0.5_O_2_ (Fe/CAZO), was investigated for the RWGS reaction with and without an electric field at 423 K. Fe/CAZO exhibited high CO_2_ conversion and CO selectivity (ca. 100%), with its catalytic performance being maintained for at least 8 h. The apparent activation energy was estimated to be 5.9 kJ mol^−1^ under an electric field, which was much lower than that without an electric field (61.4 kJ mol^−1^). The application of an electric field to catalysts enables low-temperature selective CO_2_ conversion to proceed even though no activity was observed in the conventional RWGS reaction without an electric field (0 mA). This is the first report showing that the RWGS reaction proceeds over Fe-based catalysts without platinum group metals (PGM) at low temperatures below 500 K. These results will lead to the creation of a new CO_2_ recycling technology that uses an inexpensive/abundant Fe-based catalyst and unused low-temperature waste heat.

## Experimental

### Catalyst preparation

CeO_2_ was supplied by the Catalyst Society of Japan (JRC-CEO-1). Ce_0.5_Zr_0.5_O_2_ (denoted as CZO) and Ce_0.4_Al_0.1_Zr_0.5_O_2_ (denoted as CAZO) were prepared using a complex polymerization method based on a synthetic procedure presented in the literature as follows^27^. Citric acid monohydrate (FUJIFILM Wako Pure Chemical Co.) and ethylene glycol (FUJIFILM Wako Pure Chemical Co.) were dissolved in 200 mL of distilled water and stirred. Then, stoichiometric amounts of Ce(NO_3_)_3_⋅6H_2_O (FUJIFILM Wako Pure Chemical Co.), ZrO(NO_3_)_2_⋅2H_2_O (FUJIFILM Wako Pure Chemical Co.) and Al(NO_3_)_3_⋅9H_2_O (FUJIFILM Wako Pure Chemical Co.) were added to the solution and stirred. The molar ratios of metal, citric acid monohydrate and ethylene glycol were 1:3:3. The prepared solution was heated on a hot plate at 523 K with stirring to remove water and then dried in an oven at 343 K overnight. The obtained powder was calcined at 773 K for 5 h (5 K min^−1^).

Using Fe(NO_3_)_3_⋅9H_2_O (FUJIFILM Wako Pure Chemical Co.), Co(NO_3_)_2_⋅6H_2_O (FUJIFILM Wako Pure Chemical Co.) or Ni(NO_3_)_2_·6H_2_O (FUJIFILM Wako Pure Chemical Co.) as precursors, 10 wt% Fe, Co or Ni, respectively, was supported on the prepared supports by an impregnation method based on a synthetic procedure presented in the literature as follows^28^. Each of the support powders was dispersed in 20 mL of distilled water and stirred for 2 h at room temperature *in vacuo*. Then, 20 mL of an aqueous solution of the metal precursor was added to the support-powder-dispersed solution and stirred for 2 h. The mixed solution was heated on a hot plate at 523 K with stirring to remove water and then dried in an oven at 343 K overnight. The obtained powder was calcined at 773 K for 2 h (5 K min^−1^).

The crystalline structure of the prepared catalysts was characterized by powder X-ray diffraction (XRD; X’part-PRO; PANalytical), which was performed at 45 kV and 40 mA using Cu-Kα radiation. Diffractograms were taken at 2*θ* angles of 3–75° with a step size of 0.01°.

### Activity test

**Figure 7:**
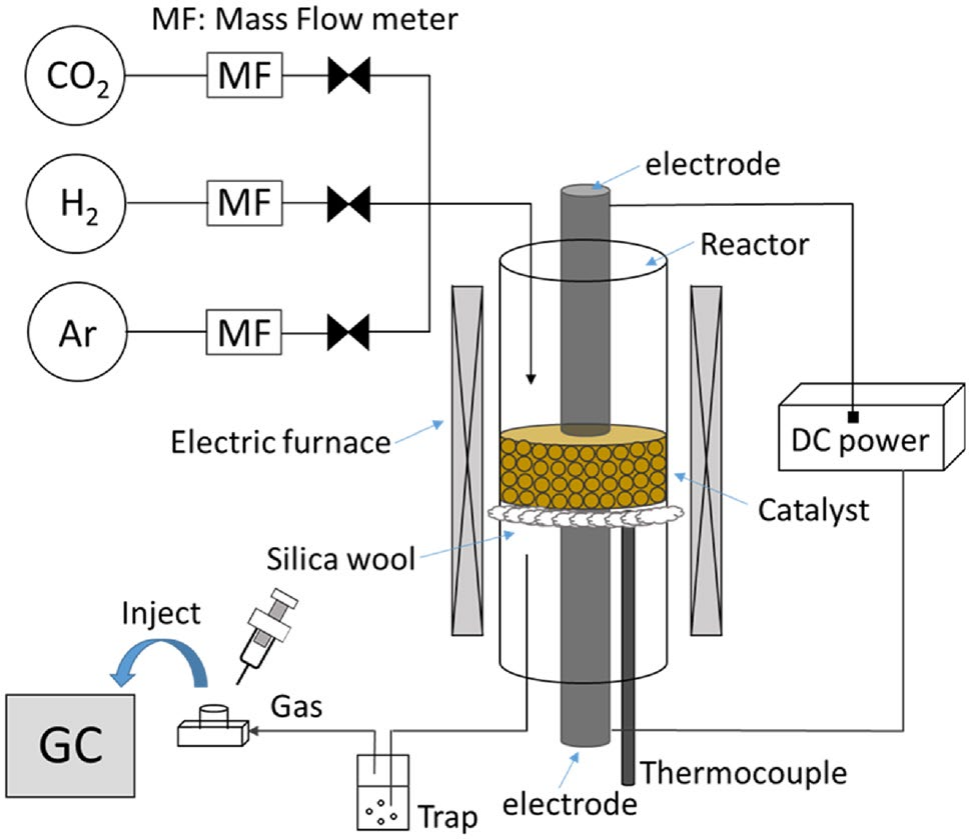
Schematic diagram of the reaction apparatus.

Catalytic activity tests with or without an electric field were conducted using a fixed-bed flow-type reactor with a quartz tube (6.0 mm i.d.), as shown in Fig. 7. The catalysts were sieved to 250–500 µm, and 100 mg of each sample was charged into a reaction tube. For pretreatment, the catalyst was reduced at 773 K for 2 h under a H_2_/Ar gas flow (H_2_:Ar = 1:2, 75 mL min^−1^). After the reduction, the furnace temperature was lowered to 423 K. The composition of the reactant feed gas was CO_2_:H_2_:Ar = 1:1:2 (100 mL min^−1^). Two stainless-steel electrodes (2.0 mm o.d.) were inserted into each end of the catalyst bed to apply a direct current using a DC power supply. The catalyst bed temperature was directly measured using a thermocouple, which was inserted into the bottom side of the catalyst bed. After removal of the produced water using a cooling trap, product gases, including CO_2_, CO, and CH_4_, were analyzed using a gas chromatograph-flame ionization detector (GC-FID, GC-2014; Shimadzu Corp.) equipped with a Porapak Q packed column and a methanizer (MTN-1; Shimadzu Corp.). The CO_2_ conversion and CO selectivity were calculated by the following equations (Eqs. 2 and 3, respectively):

2 $${{\text{CO}}}_{2}\text{ conversion }\left(\text{\%}\right)=({F}_{{\text{CO}},{\text{out}}}+{F}_{{{\text{CH}}}_{4},{\text{out}}})/{F}_{{{\text{CO}}}_{2},{\text{in}}} \times 100$$

3 $$\text{CO selectivity }\left(\text{\%}\right)= {F}_{{\text{CO}},{\text{out}}}/{(F}_{{\text{CO}},{\text{out}}}+{F}_{{{\text{CH}}}_{4},{\text{out}}})\times 100$$

### Supplementary Information

Supplementary Information 1.

Supplementary Information 2.

## Data Availability

Data is provided within the manuscript or supplementary information files.

## References

[1] Cox PM, Betts RA, Jones CD, Spell SA, Totterdell IJ (2000). Acceleration of global warming due to carbon-cycle feedbacks in a coupled climate model. Nature.

[2] Rogelj J, den Elzen M, Höhne N, Fransen T, Fekete H, Winkler H, Schaeffer R, Sha F, Riahi K, Meinshausen M (2016). Paris Agreement climate proposals needs a boost to keep warming well below 2 °C. Nature.

[3] Davis SJ, Caldeira K, Matthews HD (2010). Future CO_2_ emissions and climate change from existing energy infrastructure. Science.

[4] Tsubaki N, Wang Y, Yang G, He Y (2023). Rational design of novel reaction pathways and tailor-made catalysts for value-added chemicals synthesis from CO_2_ hydrogenation. Bull. Chem. Soc. Jpn..

[5] Yamada K, Ogo S, Yamano R, Higo T, Sekine Y (2020). Low-temperature conversion of carbon dioxide to methane in an electric field. Chem. Lett..

[6] Kwak JH, Kovarik L, Szanyi J (2013). Heterogeneous catalysis on atomically dispersed supported metal: CO_2_ reduction on multifunctional Pd catalysts. ACS Catal..

[7] Zhu J, Zhang G, Li W, Zhang X, Ding F, Song C, Guo X (2020). Deconvolution of the particle size effect on CO_2_ hydrogenation over iron-based catalysts. ACS Catal..

[8] Liu H-X, Li S-Q, Wang W-W, Yu W-Z, Zhang W-J, Ma C, Jia C-J (2022). Partially sintered copper-ceria as excellent catalyst for the high-temperature reverse water gas shift reaction. Nat. Commun..

[9] Zhu Y, Zheng J, Ye J, Cui Y, Koh K, Kovarik L, Camaioni DM, Fulton JL, Truhlar DG, Neurock M, Cramer CJ, Gutiérrez OY, Lercher JA (2020). Copper-zirconia interfaces in UiO-66 enable selective catalytic hydrogenation of CO_2_ to methanol. Nat. Commun..

[10] Lam E, Larmier K, Wolf P, Tada S, Safonova OV, Coperet C (2018). Isolated Zr surface sites on silica promote hydrogenation of CO_2_ to CH_3_OH in supported Cu catalysts. Chem. Soc..

[11] Ting KW, Toyao T, Siddiki SMAH, Shimizu K (2019). Low-temperature hydrogenation of CO_2_ to methanol over heterogeneous TiO_2_-supported Re catalysts. ACS Catal..

[12] Zhu J, Ciolca D, Liu L, Parastaev A, Kosinov N, Hensen EJM (2021). Flame synthesis of Cu/ZnO-CeO_2_ catalysts: Synergistic metal-support interactions promote CH_3_OH selectivity in CO_2_ hydrogenation. ACS Catal..

[13] Xu M, Cao C, Xu J (2022). Understanding kinetically interplaying reverse water-gas shift and Fischer-Tropsch synthesis during CO_2_ hydrogenation over Fe-based catalysts. Appl. Catal. A.

[14] Li J, He Y, Tan L, Zhang P, Peng X, Oruganti A, Yang G, Abe H, Wang Y, Tsubaki N (2018). Integrated tunable synthesis of liquid fuels via Fischer-Tropsch technology. Nat. Catal..

[15] Ogo S, Sekine Y (2017). Catalytic reaction assisted by plasma or electric field. Chem. Rec..

[16] Torimoto M, Murakami K, Sekine Y (2019). Low-temperature heterogeneous catalytic reaction by surface protonics. Bull. Chem. Soc. Jpn..

[17] Oshima K, Shinagawa T, Nogami Y, Manabe R, Ogo S, Sekine Y (2014). Low temperature catalytic reverse water gas shift reaction assisted by an electric field. Catal. Today.

[18] Yamano R, Ogo S, Nakano N, Higo T, Sekine Y (2023). Non-conventional low-temperature reverse water-gas shift reaction over highly dispersed Ru catalyst in an electric field. EES Catal..

[19] Motomura A, Nakaya Y, Sampson C, Higo T, Torimoto M, Tsuneki H, Furukawa S, Sekine Y (2022). Synergistic effects of Ni-Fe alloy catalysts on dry reforming of methane at low temperatures in electric field. RSC Adv..

[20] Nakano N, Torimoto M, Sampei H, Yamashita R, Yamano R, Saegusa K, Motomura A, Nagakawa K, Tsuneki H, Ogo S, Sekine Y (2022). Elucidation of the reaction mechanism on dry reforming of methane in an electric field by in-situ DRIFTs. RSC Adv..

[21] Dazza YA, Kuhn JN (2016). CO_2_ conversion by reverse water gas shift catalysis: comparison of catalysts, mechanisms and their consequences for CO_2_ conversion to liquid fuels. RSC Adv..

[22] Kim G, Shin S, Choi Y, Kim J, Kim G, Kim K, Lee H (2022). Gas-permeable iron-doped ceria shell on Rh nanoparticles with high activity and durability. JACS Au.

[23] Liu Y, Zhang G, Liu S, Zhu J, Liu J, Wang J, Li R, Wang M, Fu Q, Hou S, Song C, Guo X (2022). Promoting n-butane dehydrogenation over PtMn/SiO_2_ through structural evolution induced by a reverse water-gas shift reaction. ACS Catal..

[24] Gu M, Dai S, Qiu R, Ford ME, Cao C, Wachs IE, Zhu M (2021). Structure-activity relationships of copper-and potassium-modified iron oxide catalysts during reverse water-gas shift reaction. ACS Catal..

[25] Alvarez-Galvan C, Lustemberg PG, Oropeza FE, Bachiller-Baeza B, Ospina MD, Herranz M, Cebollada J, Collado L, Campos-Martin JM, de la Pena-O’Shea VA, Alonso JA, Ganduglia-Pirovano MV (2022). Highly active and stable Ni/La-doped ceria material for catalytic CO_2_ reduction by reverse water-gas shift reaction. ACS Appl. Mater. Interfaces.

[26] Zhang X, Zhu X, Lin L, Zhang M, Liu X, Wang X, Li Y, Shi C, Ma D (2016). Highly dispersed copper over β-Mo_2_C as an efficient and stable catalyst for the reverse water gas shift (RWGS) reaction. ACS Catal..

[27] Sakai R, Murakami K, Mizutani Y, Tanaka Y, Hayashi S, Ishikawa A, Higo T, Ogo S, Tsuneki H, Nakai H, Sekine Y (2020). Agglomeration suppression of a Fe-Supported catalyst and its utilization for low-temperature ammonia synthesis in an electric field. ACS Omega.

[28] Torimoto M, Ogo S, Harjowinoto D, Higo T, Seo JG, Furukawa S, Sekine Y (2019). Enhanced methane activation on diluted metal–metal ensembles under an electric field: breakthrough in alloy catalysis. Chem. Commun..

