# Supplementary material for: Efficient reverse water gas shift reaction at low temperatures over an iron supported catalyst under an electric field
Source: Sci Rep. 2024 May 3;14:10216. doi: 10.1038/s41598-024-61017-2 (PMC11068772; doi:10.1038/s41598-024-61017-2)
Supplement: Supplementary file 2 — Supplementary Information 2. [file 41598_2024_61017_MOESM2_ESM.pdf]

Supporting Information

Efficient reverse water gas shift reaction at low temperatures over an iron supported catalyst under an electric field

Masaki Yamaoka[a], Keidai Tomozawa[a], Koki Sumiyoshi[a], Tadaharu Ueda[a,b,c], Shuhei Ogo[a,b,*]

Affiliation:

[a] Department of Marine Resources Science, Faculty of Agriculture and Marine Science, Kochi University, Nankoku, Kochi 783-8502, Japan

[b] Center for Advanced Marine Core Research, Kochi University, Nankoku, Kochi 783-8502, Japan

[c] MEDi Center, Kochi University, Kochi 780-0842, Japan

***Corresponding author:** Shuhei Ogo (ogo@kochi-u.ac.jp)

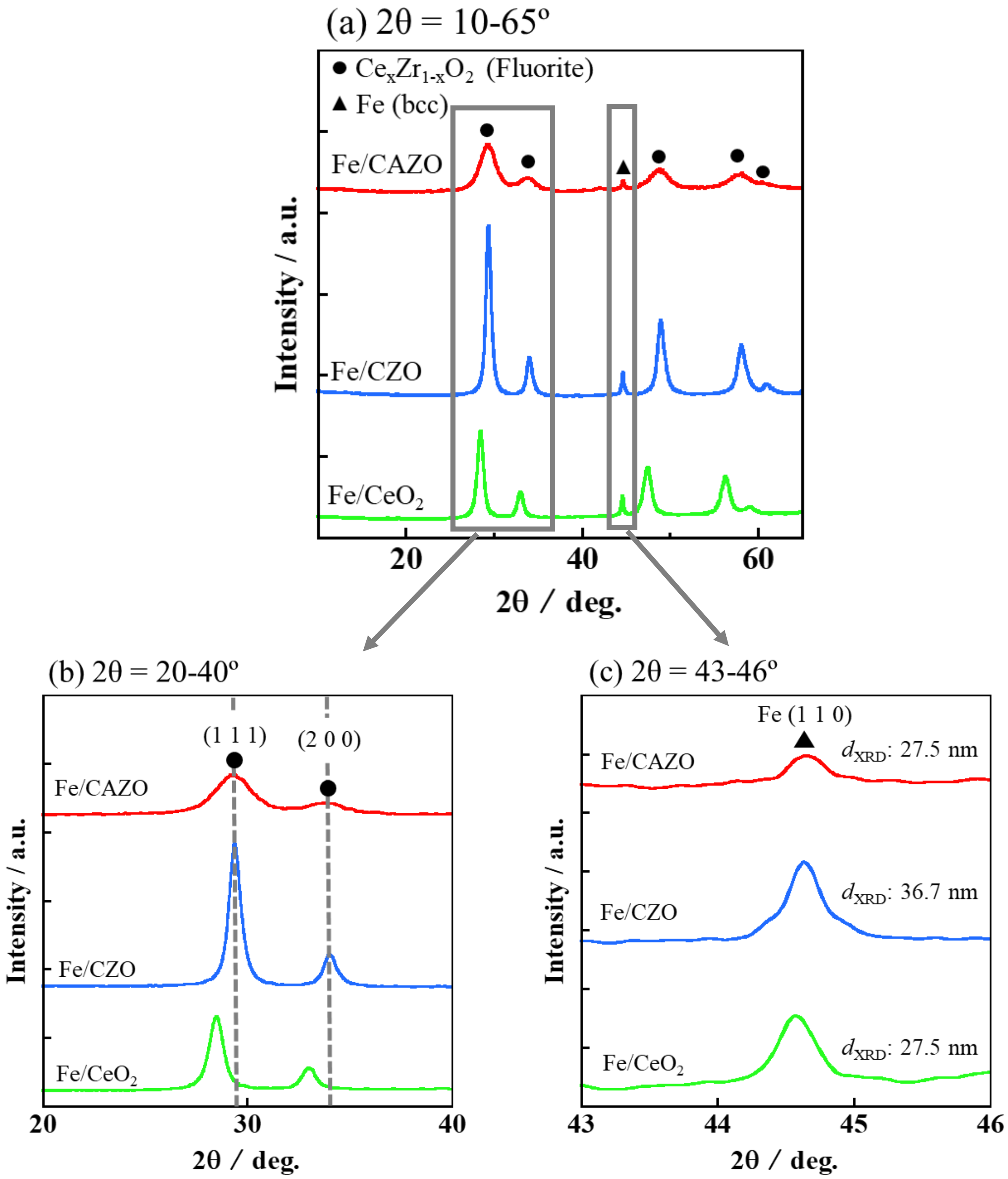

Fig. S1 XRD patterns of the10 wt% Fe/support catalysts after the RWGS reaction under an electric field.

Periodic operation test: CO_2-H_2 alternating supply

CO_2-H_2 alternating supply activity tests were conducted using a fixed-bed flow-type reactor with a quartz tube (6.0 mm i.d.). The Fe/CAZO catalyst was sieved to 250-500 µm, and 100 mg of it was charged into the reaction tube. For pretreatment, the catalyst was reduced at 773 K for 2 h under a H_2/Ar gas flow (H_2:Ar = 1:2, total flow rate = 75 mL min^{-1}). After the reduction, the furnace temperature was lowered to 473 K, followed by an Ar purge (50 mL min^{-1}). In the first step, CO_2+Ar gas (CO_2:Ar = 1:2, total flow rate = 75 mL min^{-1}) was supplied to the reactor under an electric field (5 mA) for 17 min, and the produced CO gas was analyzed by the GC every 5 min. In the second step, the residual CO_2 in the gas phase of the reactor was removed by an Ar purge (50 mL min^{-1}) for 10 min. In the third step, H_2+Ar gas (H_2:Ar = 1:2, total flow rate = 75 mL min^{-1}) was supplied to the reactor under an electric field (5 mA) for 17 min, and the produced CO gas was analyzed by the GC every 5 min. In the final step, an Ar purge was applied for 10 min to remove all residual gases. These steps were repeated for two cycles.

Figure S2(a) shows the integrated amount of CO formed during CO_2 or H_2 supply over the Fe/CAZO catalyst under an electric field (5 mA) at 473 K. In the 1st cycle, CO was produced under the electric field when CO_2 was supplied after H_2 reduction. In contrast, no CO formation was observed when H_2 was supplied even if an electric field was applied. Similar to the 1st cycle, CO was produced only when CO_2 was supplied under the electric field in the 2nd cycle. These results suggested that the RWGS reaction over the Fe/CAZO catalyst under an electric field proceeded through a redox mechanism (Fig. S2(b)) in which CO was produced by the reaction between CO_2 and a lattice oxygen vacancy, and this vacancy was formed by the reaction between H_2 and a lattice oxygen.

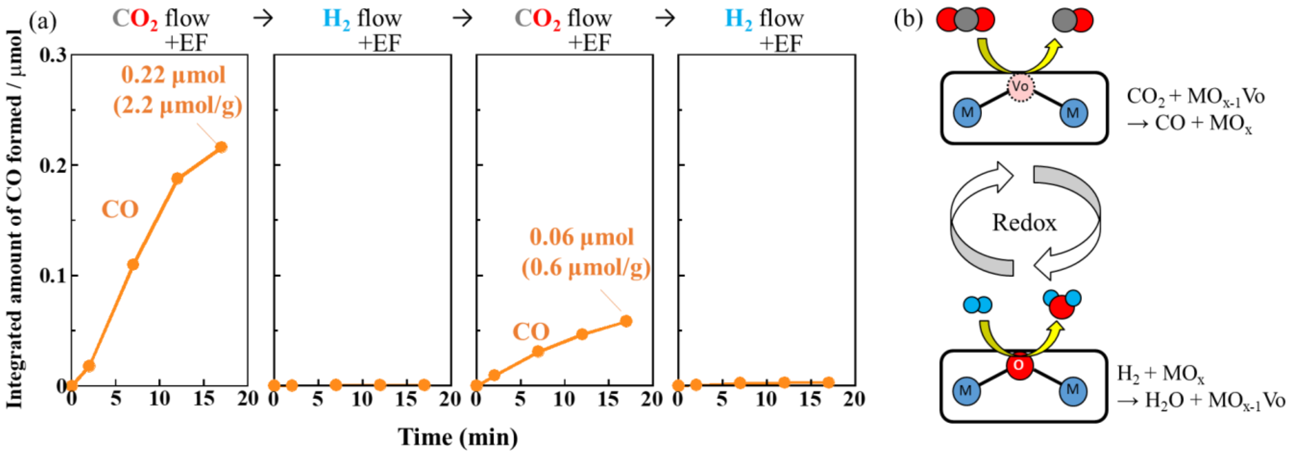

Fig. S2 (a) Integrated amount of CO formed during CO_2 or H_2 supply over the Fe/CAZO catalyst under an electric field (5 mA) at 473 K after H_2 pretreatment at 773 K. (b) Possible RWGS reaction mechanism under an electric field.

Then, to clarify the effect of the electric field on the redox activity of the Fe/CAZO catalyst, the integrated amount of CO formed was investigated during CO_2 or H_2 supply over the Fe/CAZO catalyst without an electric field (0 mA) at 473 K (Fig. S3). Without an electric field, the integrated amount of CO formed during CO_2 supply was significantly reduced, indicating that the application of an electric field to the catalyst is needed for the redox reaction to proceed using a lattice oxygen vacancy at low temperatures below 500 K. In other words, an electric field promotes the redox reaction, as shown in Fig. S2(b).

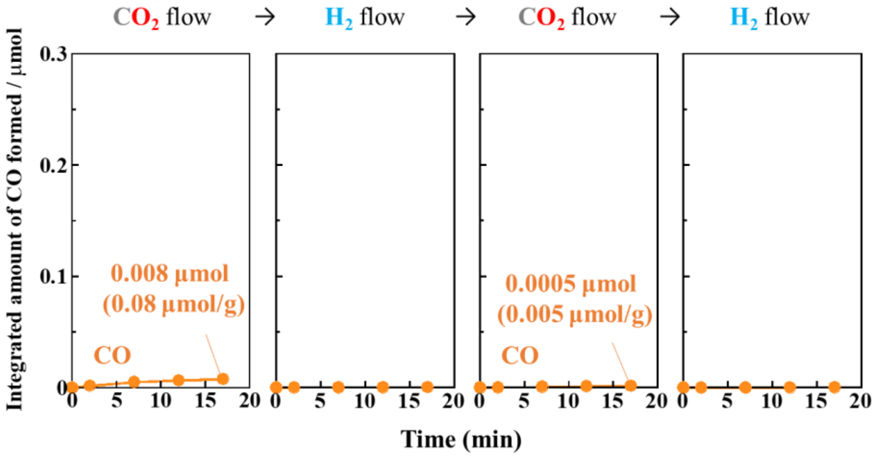

Fig. S3 Integrated amount of CO formed during CO_2 or H_2 supply over the Fe/CAZO catalyst without an electric field (0 mA) at 473 K after H_2 pretreatment at 773 K.

Catalytic stability test: Daily startup and shutdown operation

The catalytic stability of the Fe/CAZO catalyst was evaluated during daily startup and shutdown operations. Before the stability test, the catalyst was reduced under H_2 flow at 773 K for 2 h. The catalytic stability tests were performed under an electric field (5 mA) at 423 K for 8.5 h each day. After the 8.5 h durability test, the electric field was turned off, and the catalyst was purged with Ar gas at 423 K overnight.

Figure S4 shows the CO_2 conversion and CO selectivity variations with time elapsed on stream over the Fe/CAZO catalyst under an electric field during the daily startup and shutdown operation. The CO_2 conversion and CO selectivity remained constant during three days of operation, although some fluctuations were observed, indicating that the Fe/CAZO catalyst should be highly stable.

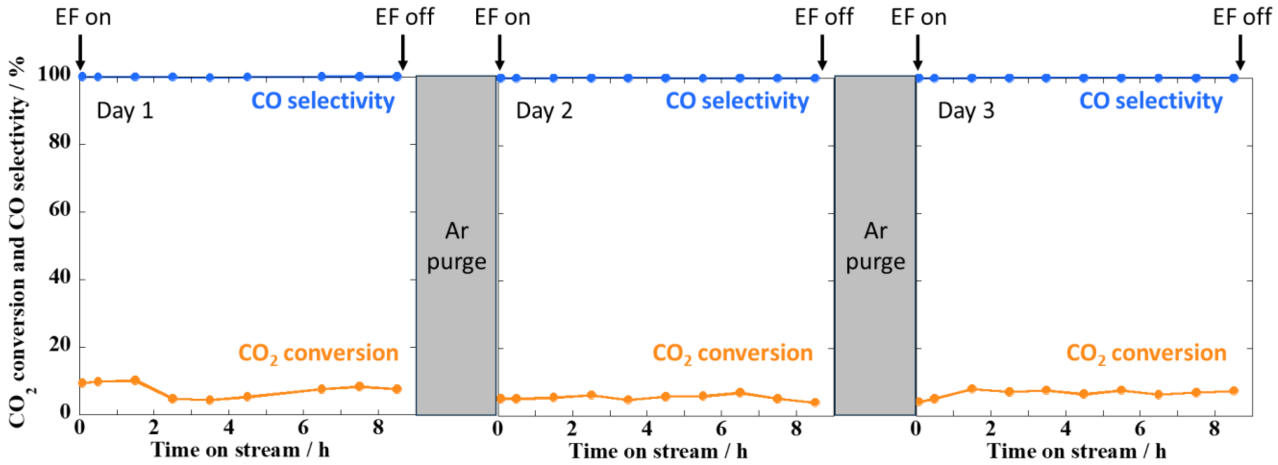

Fig. S4 Catalytic stability during the RWGS reaction over the Fe/CAZO catalyst under an electric field in the daily startup and shutdown operation.

Furnace temperature: 423 K; catalyst weight: 100 mg; input current: 5.0 mA; gas composition (%): CO_2:H_2:Ar = 25:25:50; total gas flow rate: 100 mL min^{-1}.

Raw data for production of Figures

For Fig. 1

Metal	Current / mA	Voltage / kV	CO_2 conversion / %	CO yield / %	CH_4 yield / %
Fe	3	0.45	8.0	8.0	0.0
	5	0.31	11.5	11.5	0.0
	7	0.29	15.0	15.0	0.0
Co	3	0.42	14.6	7.3	7.3
	5	0.31	18.2	9.5	8.7
	7	0.33	20.7	12.7	8.1
Ni	3	0.46	19.2	6.3	12.9
	5	0.27	18.2	7.1	11.1
	7	0.23	20.3	7.9	12.3

For Fig. 2

Fe loading amount / wt%	Current / mA	Voltage / kV	CO_2 conversion / %	CO selectivity / %
0	1	Discharge	0.0	-
	3	Discharge	0.0	-
	5	Discharge	0.0	-
5	1	0.35	0.3	100
	3	0.40	5.1	99.9
	5	0.27	6.4	99.9
10	1	0.66	2.4	99.4
	3	0.45	8.0	99.7
	5	0.31	11.5	99.8
15	1	0.62	2.5	98.7
	3	0.47	8.8	99.7
	5	0.31	12.0	99.9

For Fig. 3

Catalyst	Current / mA	Electric power / W	CO_2 conversion / %
Fe/CeO_2	3	0.36	0.3
	5	0.50	0.9
	7	0.63	1.6
Fe/CZO	3	0.90	4.7
	5	1.40	8.5
	7	1.54	10.1
Fe/CAZO	3	1.35	8.0
	5	1.55	11.5
	7	2.03	15.0

For Fig. 4

Current / mA	Temperature / K	CO_2 conversion / %
0	432	0.0
	485	0.2
	537	0.7
	587	2.5
	637	5.0
	688	7.7
	737	10.9
	787	14.9
3	450	8.1
	500	9.9
	550	11.4
	600	12.4
	648	15.1
	694	16.3
	746	22.4
	791	27.4
5	475	11.6
	532	12.8
	582	13.3
	631	15.4
	677	18.7
7	475	13.8
	528	13.2
	577	15.8
	627	17.0
	675	19.9
	725	24.9
	774	28.1
equilibrium	373	1.6
	423	3.4
	473	6.1
	523	9.5
	573	13.5
	623	17.8
	673	22.2
	723	26.6
	773	30.7
	823	34.6

For Fig. 5

Current / mA	1000/T / K^{-1}	ln(r_{CO} / µmol sec^{-1})
0	2.31	-5.49
	2.06	-3.52
	1.86	-2.11
	1.70	-0.77
	1.57	-0.07
	1.45	0.36
	1.36	0.71
	1.27	1.02
5	2.11	0.77
	1.88	0.88
	1.72	0.92
	1.58	1.05
	1.48	1.25

For Fig. 6

Time on stream / h	CO_2 conversion / %	CO selectivity / %
0.0	9.7	100
0.5	10.4	100
1.0	9.5	100
1.5	9.9	100
2.0	9.1	100
2.5	8.2	100
3.0	7.7	100
3.5	8.7	100
4.0	8.2	100
4.5	8.6	100
5.0	8.7	100
5.5	8.4	100
6.0	9.1	100
6.5	9.7	100
7.0	8.2	100
7.5	9.6	100
8.0	10.0	100

For Fig. S1

Another Excel file is attached.

For Fig. S2

Operation	Time on stream / h	Integrated amount of CO formed / μmol
CO_2 flow-1	0	0.000
	2	0.018
	7	0.110
	12	0.188
	17	0.216
H_2 flow-1	0	0.000
	2	0.000
	7	0.000
	12	0.000
	17	0.000
CO_2 flow-2	0	0.000
	2	0.009
	7	0.031
	12	0.047
	17	0.059
H_2 flow-2	0	0.000
	2	0.001
	7	0.002
	12	0.003
	17	0.003

For Fig. S3

Operation	Time on stream / h	Integrated amount of CO formed / μmol
CO_2 flow-1	0	0.000
	2	0.002
	7	0.005
	12	0.007
	17	0.008
H_2 flow-1	0	0.000
	2	0.000
	7	0.000
	12	0.000
	17	0.001
CO_2 flow-2	0	0.000
	2	0.000
	7	0.001
	12	0.001
	17	0.002
H_2 flow-2	0	0.000
	2	0.000
	7	0.000
	12	0.000
	17	0.000

For Fig. S4

Day 1

Time on stream / h	CO_2 conversion / %	CO selectivity / %
0.08	9.4	100
0.5	9.8	99.9
1.5	10.2	99.9
2.5	4.7	99.9
3.5	4.3	99.8
4.5	5.3	99.9
6.5	7.6	100
7.5	8.3	100
8.5	7.6	100

Day 2

Time on stream / h	CO_2 conversion / %	CO selectivity / %
0.08	5.0	100
0.5	5.0	99.9
1.5	5.4	100
2.5	6.2	100
3.5	4.7	100
4.5	5.7	100
5.5	5.8	99.9
6.5	6.8	100
7.5	5.1	99.9
8.5	3.9	99.9

Day 3

Time on stream / h	CO_2 conversion / %	CO selectivity / %
0.08	4.1	100
0.5	5.1	99.9
1.5	7.9	100
2.5	7.1	100
3.5	7.5	100
4.5	6.4	100
5.5	7.6	100
6.5	6.3	100
7.5	6.9	100
8.5	7.3	100
